# Technology-Enriched High-Fidelity Simulation in Nursing Education: Learning Effectiveness and Mediating Model

**DOI:** 10.1007/s40670-026-02842-0

**Published:** 2026-07-31

**Authors:** Cochava Sharon, Inbal Halevi Hochwald, Gizell Green, Alena Lochmannová

**Affiliations:** 1 Department of Nursing, Sha’arei Mishpat College, Hod HaSharon, Israel; 2 Nursing department, Max Stern Yezreel Valley College, D.N. Emek, 1930600 Israel; 3 Department of Crisis Management, Police Academy of the Czech Republic in Prague, Lhotecká 559/7, Prague 12, 143 00 Czech Republic

**Keywords:** Nursing Education, Simulation Training, High Fidelity Simulation Training, Clinical Competence, Problem Solving, Debriefing

## Abstract

**Background:**

Despite extensive research documenting high-fidelity simulation benefits, limited evidence exists regarding the effectiveness of simulation explicitly enriched with clinical technologies or the mechanisms through which simulation components influence learning outcomes.

**Aims:**

To evaluate nursing students’ learning effectiveness across multiple dimensions following high-fidelity simulation enriched with clinical technologies (HFSECT) and to test whether course arrangement, equipment resources, and clinical ability mediate the relationship between debriefing quality and problem-solving.

**Methods:**

Eighty-one third-year undergraduate nursing students participated in a quasi-experimental one-group pretest–posttest study involving HFSECT with dual-component debriefing. The Simulation Learning Effectiveness Inventory assessed seven dimensions: course arrangement, equipment resources, debriefing, clinical ability, confidence, problem-solving, and collaboration. Learning satisfaction was assessed separately. A parallel mediation analysis was conducted using the PROCESS macro with 5,000 bootstrap samples.

**Results:**

Significant improvements occurred in six dimensions: course arrangement, equipment resources, debriefing, problem-solving, clinical ability, and confidence, with substantial satisfaction gains. Collaboration showed no significant change. Mediation analysis showed a significant indirect association through clinical ability, whereas the indirect associations through course arrangement and equipment resources were not significant. The direct association between debriefing and problem-solving remained significant.

**Conclusions:**

HFSECT was associated with improvements in nursing students’ perceived learning effectiveness and satisfaction. Clinical ability mediated the association between debriefing and problem-solving, suggesting that debriefing may support problem-solving partly through clinical ability.

## Introduction

The contemporary healthcare landscape is characterized by unprecedented complexity and technological advancement [1–4]. The demands placed on nursing education and service delivery are also changing [5]. These developments require nurses to maintain current knowledge and competencies [6]. Nursing educators therefore need to provide students with opportunities to develop higher-order thinking and clinical competence. Simulation-based learning (SBL) offers a safe setting in which students can practice clinical skills and apply theoretical knowledge without placing patients at risk [7–9]. Simulation has been associated with improvements in clinical knowledge and practical skills [10–12]. It may also support critical thinking [13] and the assessment of clinical competence [14].

High-fidelity simulation (HFS) uses full-scale computerized patient simulators to reproduce realistic clinical manifestations and scenarios [15]. Compared with other teaching methods, HFS has been associated with better knowledge and performance outcomes among nursing students [16], while higher mannequin fidelity may improve immediate outcomes across nursing and related healthcare education [17]. Li et al. [18] also reported larger gains in knowledge and skills, but no clear advantage for critical thinking or for students’ confidence and satisfaction.

Digital systems and clinical technologies are increasingly embedded in nursing practice [19] and students encounter digital patient systems during clinical placements [20]. Their inclusion in simulation may make training more consistent with current clinical practice. We use the term high-fidelity simulation enriched with clinical technologies (HFSECT) for this approach.

Yet gaps remain in the technological preparation of undergraduate nursing students. In le Roux et al. [21], only 15.2% reported previous training in nursing informatics or healthcare technology, while curriculum mapping identified no training in information management. Opportunities to work with technology during nursing education may therefore be insufficient. Simulation offers one way to provide this experience and has been used to assess students’ perceived competence in clinical behaviors and skills [22].

Simulation may also influence students’ confidence and satisfaction. Kang et al. [23] found that both outcomes differed across educational formats, while Arrogante et al. [24] reported high satisfaction following clinical simulation. The frequency of simulation exposure may also matter. Cummings and Connelly [25] associated greater exposure to simulation with higher confidence, and Hung et al. [26] reported improvements in perceived competence and learning satisfaction across repeated exposures. These findings suggest that simulation may be more useful when incorporated at several points in the nursing curriculum rather than delivered as a single exercise.

High-fidelity simulation has been associated with improved knowledge and performance among nursing students [16], while benefits have also been reported for skill development and collaboration [18]. The Simulation Learning Effectiveness Inventory distinguishes seven dimensions of simulation learning: course arrangement, equipment resources, debriefing, clinical ability, confidence, problem-solving, and collaboration [27]. The present study examines how selected dimensions are related within HFSECT by testing whether course arrangement, equipment resources, and clinical ability mediate the association between debriefing and problem-solving.

**Fig. 1:**
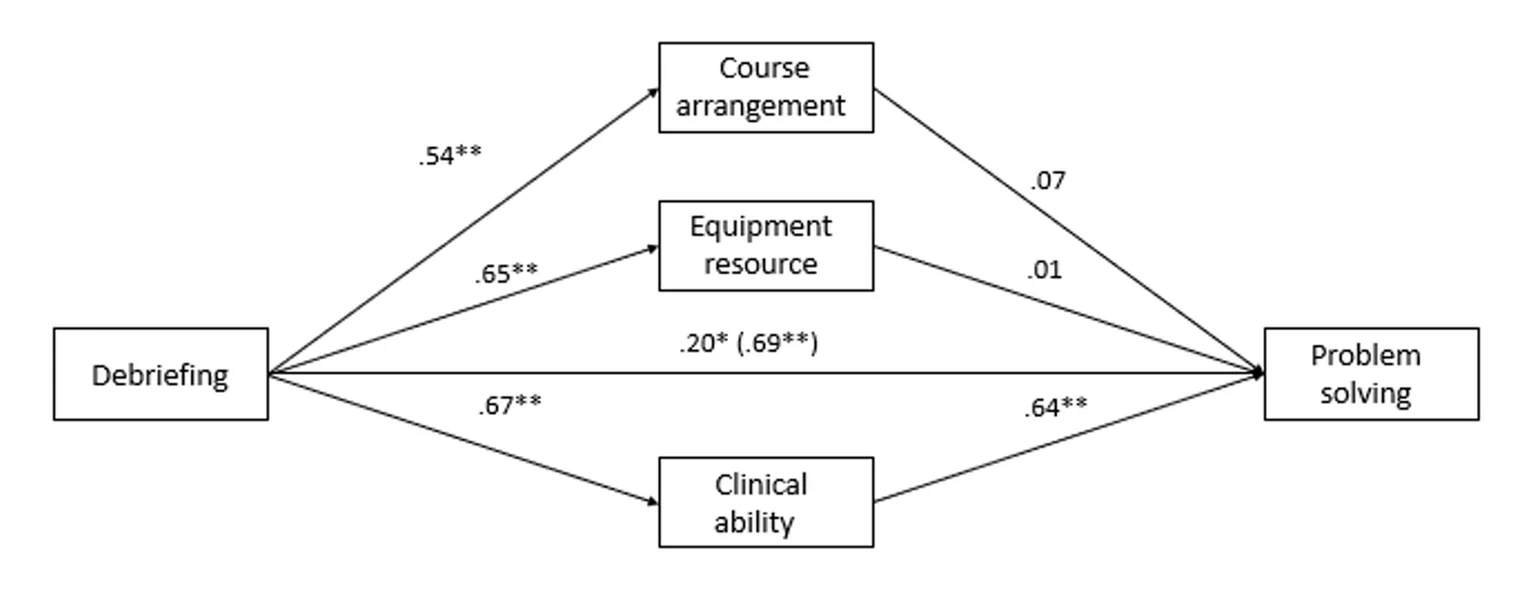
Parallel mediation model. Values are standardized regression coefficients. The coefficient outside parentheses represents the direct effect, whereas the coefficient in parentheses represents the total effect. * *p* <.05; **p <.01

Accordingly, the research aims are as follows:

1. To evaluate nursing students’ scores on the Simulation Learning Effectiveness Inventory (SLEI) and their overall learning satisfaction before and after participation in HFSECT. We hypothesized that all SLEI dimensions and learning satisfaction would improve following the intervention.
2. To examine bivariate associations among SLEI dimensions and learning satisfaction before the intervention (T1) and after the intervention (T2).
3. To test a parallel mediation model examining whether course arrangement, equipment resources, and clinical ability mediate the relationship between debriefing quality and problem-solving following HFSECT (see Fig. 1).

## Materials and Methods

### Design

This study employed a quasi-experimental, one-group pretest-posttest repeated-measures design to examine changes in nursing students’ learning outcomes following HFSECT. Randomization was not feasible within the institutional curriculum structure. The repeated-measures design therefore enabled within-subject comparisons.

### Setting

The study was conducted at a simulation laboratory within an academic nursing institution in Israel. The facility was equipped with state-of-the-art resources specifically designed to support technology-enriched simulation learning, including: (a) high-fidelity patient simulators capable of replicating diverse physiological responses and medical conditions; (b) contemporary clinical technologies mirroring those used in modern healthcare settings, including vital signs monitors, barcode medication systems, and electronic documentation platforms; (c) configurable clinical environments designed to authentically replicate various healthcare scenarios; and (d) dedicated debriefing rooms equipped with audio-visual recording and playback systems to facilitate reflective learning and comprehensive feedback sessions.

### Participants and Sampling

#### Eligibility Criteria

Inclusion criteria were (a) enrollment in the four-year BSN program, (b) completion of the theoretical component of the Adult Nursing course, and (c) voluntary consent to participate. Students who had not completed the prerequisite course or were enrolled in other academic years were excluded.

#### Sample Size and Recruitment

An a priori power analysis using G*Power 3.1.9.4 indicated that 54 participants were required for a repeated-measures design assuming f = 0.25, α = 0.05, power = 0.80, two measurements, and *r* =.50 between measurements. Using convenience sampling, 81 third-year undergraduate nursing students meeting the eligibility criteria voluntarily consented to participate in the study, exceeding the minimum required sample size and providing adequate statistical power for the planned analyses.

### Simulation Intervention Conceptual Framework

The HFSECT intervention was designed and implemented based on Jeffries’s Simulation Framework [28], which comprises student factors, teacher factors, educational practices, simulation design characteristics, and outcomes. The framework guided the development of four clinical scenarios situated in technology-rich environments that students were required to navigate to provide safe and effective patient care. The constructivist learning approach embedded within this framework enabled students to actively construct knowledge as they engaged with realistic clinical situations requiring integration of theoretical knowledge and technological competencies.

### Simulation Intervention

Three days before the simulation, participants attended a 60-minute pre-briefing led by the simulation instructor. The instructor explained the intervention structure, clarified the objectives, introduced the setting and patient scenario, and reviewed the patient history, anticipated complications, and clinical techniques to be practiced.

During the simulation phase, training was delivered over two days, comprising four scenario sessions lasting 45 min each, followed by a 30-minute debriefing after each scenario. During the simulation, students practiced core nursing skills within complex scenarios while using clinical technologies such as thermometers, vital signs monitors, pulse oximeters, spirometers, ECG devices, and oxygen delivery interfaces.

#### Example Scenario

##### Scenario Subject

Postoperative Care in a Surgical Unit.

##### Patient Profile

A 65-year-old patient recovering from abdominal surgery.

##### Clinical Scenario Care and Treatment

Students prepare the patient’s room to optimize positioning and comfort. They monitor body temperature and assess vital signs, including blood pressure, heart rate, and oxygen saturation, using bedside monitoring equipment. They maintain airway patency using an oropharyngeal airway when indicated and administer oxygen via a respiratory mask connected to an oxygen concentrator. Pain is managed using a patient-controlled analgesia (PCA) pump.

After each scenario, students completed two structured reflection activities. First, a facilitator-led debriefing focused on reviewing performance, clinical decision-making, and technique, with guided discussion of challenges and opportunities for improvement. Second, students completed an individual video-assisted reflection by reviewing a recording of their own performance and documenting strengths and areas for improvement, including both technical and non-technical aspects.

### Instruments

Data were collected using a self-administered questionnaire.

#### Demographic Questionnaire

A brief demographic form collected participant characteristics including age, gender, marital status, religious affiliation, and educational background.

#### Simulation Learning Effectiveness Inventory (SLEI)

The SLEI [27] is a validated 32-item self-report instrument designed to assess students’ perceptions of simulation learning effectiveness across seven dimensions: course arrangement (5 items), equipment resources (5 items), clinical ability (4 items), debriefing (5 items), problem-solving (4 items), confidence (5 items), and collaboration (4 items). Each item is rated on a 5-point Likert scale ranging from 1 (*strongly disagree*) to 5 (*strongly agree*), with higher scores indicating more positive perceptions of simulation effectiveness. Example item: “The course content was arranged adequately in terms of sequential order and depth, facilitating my learning.” The instrument demonstrated excellent internal consistency reliability in this study, with Cronbach’s alpha coefficients ranging from 0.81 (course arrangement) to 0.94 (collaboration). For this study, the SLEI was translated from English into Hebrew using forward and backward translation. Three bilingual nursing faculty members with expertise in simulation education reviewed the translated version for semantic equivalence.

#### Learning Satisfaction Measure

Overall learning satisfaction was assessed using a single-item 10-point numerical rating scale ranging from 1 (extremely dissatisfied) to 10 (extremely satisfied).

### Ethical Considerations

The study received ethical approval from the Institutional Review Board of the Faculty of Health Sciences at Ariel University (approval no. AU-HEA-GG-20190130). An independent researcher who was not part of the research team obtained written informed consent from all participants.

### Data Collection and Analysis

Data were collected at two time points: immediately before the simulation intervention (T1, pre-test) and within 24 h following completion of all simulation activities and debriefing sessions (T2, post-test). At both time points, participants completed the SLEI and the learning satisfaction measure in a quiet room separate from the simulation environment to minimize distractions and ensure confidentiality. Demographic information was collected only at T1. Questionnaire completion required approximately 15–20 min per administration. An a priori power analysis was conducted using G*Power version 3.1.9.4, as described above. Statistical analyses were performed in IBM SPSS Statistics version 29.0. Descriptive statistics, including frequencies, percentages, means, and standard deviations, were used to characterize the sample and study variables. A one-factor repeated-measures analysis of variance (ANOVA) examined changes in SLEI subscales and learning satisfaction from T1 to T2, with effect sizes reported as partial eta squared (ηp²). Pearson correlation coefficients assessed bivariate relationships among study variables at each time point. Statistical significance was set at α = 0.05 (two-tailed). The PROCESS macro for SPSS (Model 4) [29] was used to test a parallel mediation model in which course arrangement, equipment resources, and clinical ability mediated the association between debriefing and problem-solving at T2. This analysis employed ordinary least squares regression with bootstrapping (5,000 bootstrap samples) to generate bias-corrected 95% confidence intervals for indirect effects. Mediation was considered significant when the confidence interval for an indirect effect did not include zero.

## Results

The 81 participants had a mean age of 24 years (SD = 1.74). Their demographic characteristics are presented in Table 1.

**Table 1:** Frequency and percentage of background characteristics

Background Characteristics		*N* = 81	
		*n*	%
Gender	Male	4	4.9
	Female	77	95.1
Marital Status	Single	41	50.6
	Married	40	49.4
Religion	Jewish	79	97.5
	Other	2	2.5
Religiosity level	Secular	8	9.9
	Traditional	5	6.2
	Religious	67	82.7
	Ultra-Orthodox	1	1.2
Education	High school	81	100
	Bachelor’s degree	0	0

Changes in SLEI dimensions and learning satisfaction from T1 to T2 are presented in Table 2.

**Table 2:** Pre- and Post-Intervention Scores on Simulation Learning Effectiveness Inventory Subscales and Learner Satisfaction

Variable	Pre-Simulation M (SD)	Post-Simulation M (SD)	F	*p*	ηp²
SLEI Subscales					
Course arrangement	4.51 (0.72)	4.86 (0.29)	16.71	< 0.001	0.20
Equipment resources	4.46 (0.83)	4.82 (0.38)	12.81	< 0.001	0.16
Debriefing	4.38 (0.86)	4.67 (0.51)	7.70	0.007	0.10
Clinical ability	4.55 (0.68)	4.72 (0.51)	4.49	0.038	0.06
Confidence	4.45 (0.80)	4.64 (0.58)	4.22	0.044	0.06
Problem-solving	4.41 (0.81)	4.60 (0.58)	5.61	0.021	0.07
Collaboration	4.50 (0.72)	4.60 (0.64)	2.44	0.123	0.03
Learner Satisfaction	7.15 (1.98)	8.54 (1.72)	28.22	< 0.001	0.30

M = mean; SD = standard deviation; F = F-statistic from repeated measures analysis of variance; p = probability value (statistical significance); ηp² = partial eta squared (effect size measure).Effect size interpretation: ηp² values of 0.01 indicate small effect, 0.06 indicate medium effect, and 0.14 indicate large effect

Scores increased significantly in six of the seven SLEI dimensions. The largest changes were observed in course arrangement and equipment resources, while debriefing also improved. Collaboration did not change significantly. Learning satisfaction increased significantly after the intervention.

Correlations between learning satisfaction and SLEI dimensions at T1 are presented in Table 3.

**Table 3:** Pearson Correlations Between Simulation Learning Effectiveness Elements and Learner Satisfaction at Pre-Simulation (T1)

Variables	1	2	3	4	5	6	7	8
1. Learning satisfaction	—							
2. Course arrangement	0.28*	—						
3. Equipment resources	0.23	0.85**	—					
4. Debriefing	0.29*	0.85**	0.77**	—				
5. Clinical ability	0.29*	0.83**	0.87**	0.79**	—			
6. Confidence	0.32**	0.87**	0.86**	0.81**	0.93**	—		
7. Problem-solving	0.32**	0.83**	0.85**	0.80**	0.92**	0.93**	—	
8. Collaboration	0.36**	0.85**	0.87**	0.77**	0.91**	0.91**	0.89**	—

*p <.05. **p <.01

At T1, learning satisfaction was positively associated with all SLEI dimensions except equipment resources. The strongest association was observed for collaboration.

Post-intervention correlations are presented in Table 4. Learning satisfaction remained associated with problem-solving and collaboration, as well as confidence. The strongest association was observed for problem-solving.

**Table 4:** Pearson Correlations Between Simulation Learning Effectiveness Elements and Learner Satisfaction at Post-Simulation (T2)

Variables	1	2	3	4	5	6	7	8
1. Learning satisfaction	—							
2. Course arrangement	0.14	—						
3. Equipment resources	0.16	0.75**	—					
4. Debriefing	0.15	0.54**	0.65**	—				
5. Clinical ability	0.14	0.54**	0.76**	0.67**	—			
6. Confidence	0.24*	0.56**	0.67**	0.74**	0.86**	—		
7. Problem-solving	0.53**	0.40**	0.53**	0.59**	0.65**	0.74**	—	
8. Collaboration	0.30*	0.55**	0.70**	0.69**	0.83**	0.87**	0.82**	—

*p <.05. **p <.01

The parallel mediation model is presented in Fig. 1; Table 5. Debriefing significantly predicted all three proposed mediators, but only clinical ability significantly predicted problem-solving. The indirect effect through clinical ability was significant, whereas the indirect effects through course arrangement and equipment resources were not. The direct effect of debriefing remained significant, indicating partial mediation.

**Table 5:** Parallel Mediation Analysis: Direct and Indirect Effects of Debriefing on Problem-Solving Through Course Arrangement, Equipment Resources, and Clinical Ability

Path	β	B	SE	t	*p*	95% CI
Direct Effects: Debriefing → Mediators						
Debriefing → Course arrangement	0.54	0.34	0.05	5.75	< 0.001	[0.22, 0.46]
Debriefing → Equipment resources	0.65	0.53	0.06	7.67	< 0.001	[0.39, 0.67]
Debriefing → Clinical ability	0.67	0.68	0.08	8.17	< 0.001	[0.51, 0.84]
Direct Effects: Mediators → Problem-Solving						
Course arrangement → Problem-solving	0.07	0.13	0.15	0.86	0.391	[−0.17, 0.45]
Equipment resources → Problem-solving	0.01	0.02	0.16	0.14	0.889	[−0.29, 0.34]
Clinical ability → Problem-solving	0.64	0.71	0.11	6.48	< 0.001	[0.49, 0.93]
Direct Effect: Debriefing → Problem-Solving						
Debriefing → Problem-solving (c’)	0.20	0.22	0.09	2.35	0.021	[0.03, 0.40]
Indirect Effects (Bootstrapped, 5,000 samples)						
Debriefing → Course arrangement → Problem-solving	0.04	0.04	0.10	—	ns	[−0.12, 0.30]
Debriefing → Equipment resources → Problem-solving	0.01	0.01	0.17	—	ns	[−0.38, 0.34]
Debriefing → Clinical ability → Problem-solving	0.43	0.48	0.16	—	< 0.05	[0.20, 0.84]

β = standardized regression coefficient; B = unstandardized regression coefficient; *SE* standard error, *CI* confidence interval; c’ = direct effect controlling for mediators; ns = not significant (confidence interval includes zero). Indirect effects tested using bias-corrected bootstrap confidence intervals with 5,000 resamples. A significant indirect effect was indicated when the 95% CI did not include zero

## Discussion

This study examined changes in nursing students’ learning outcomes following high-fidelity simulation enriched with clinical technologies (HFSECT) and tested the pathways linking debriefing with problem-solving. Post-intervention scores were higher for course arrangement, equipment resources, debriefing quality, clinical ability, confidence, problem-solving, and learning satisfaction. Clinical ability was the only significant mediator of the association between debriefing and problem-solving.

Scores for course arrangement and equipment resources increased from T1 to T2. These improvements are noteworthy because Li et al. [30] identified course arrangement and equipment resources as comparatively lower-rated dimensions of HFS learning effectiveness among nursing students.

The findings are consistent with previous research reporting positive learning outcomes from HFS [30, 31] and video-assisted debriefing [32]. Lestander et al. [33] likewise emphasized the important role of debriefing in HFS teaching and its contribution to learning effectiveness. Li et al. [30] reported moderately high overall HFS learning effectiveness, with particularly positive experiences in debriefing, clinical ability, and problem-solving among Chinese nursing students. Participants’ clinical ability scores also increased following HFSECT.

Kaliyaperumal et al. [34] reported high levels of satisfaction and self-confidence among nursing students following simulation teaching. Learning satisfaction increased significantly following HFSECT, consistent with previous studies reporting high satisfaction among nursing students participating in simulation [24, 26, 34–38].

The HFSECT intervention was informed by Jeffries’s Simulation Framework [28] and was associated with improvements in clinical ability, problem-solving, confidence, and satisfaction. Jeffries’s framework emphasizes realistic scenario design to support experiential learning in HFS. In a cross-sectional study based on the same framework, elements of scenario-based simulation were associated with nursing students’ self-confidence and satisfaction [39]. The improvements observed following HFSECT support the continued relevance of Jeffries’s framework for technology-rich clinical education.

Clinical ability mediated the association between debriefing and problem-solving following HFSECT. Kang and Yu [40] reported that combining student self-debriefing with instructor-led debriefing improved the problem-solving process compared with instructor-led debriefing alone. Oh et al. [41] found that a debriefing protocol based on transformative learning theory resulted in higher problem-solving process scores than conventional debriefing.

Mediation analysis can help clarify the mechanisms linking predictors and outcomes in nursing research [42]. The present study applied this approach to examine whether course arrangement, equipment resources, and clinical ability mediated the association between debriefing and problem-solving following HFSECT.

## Conclusions

This study provides evidence of significant improvements in several dimensions of simulation learning following HFSECT. Critically, clinical ability was the only significant mediator of the association between debriefing and problem-solving.

These findings carry important implications for nursing education. Simulation programs should integrate contemporary clinical technologies mirroring authentic practice, implement dual-component debriefing combining facilitated discussion with video-based reflection, and focus on clinical competency consolidation to maximize problem-solving development. Although collaboration did not improve significantly, this may reflect the individual focus of the scenarios.

As healthcare becomes increasingly technology-intensive, HFSECT may help prepare students for technology-rich clinical environments. It links theoretical knowledge with simulated practice and current clinical technologies. Further research is needed to assess the retention of learning outcomes and their transfer to clinical practice.

### Limitations and Future Research Recommendations

While this study provides valuable insights into HFSECT effectiveness in nursing education, several limitations warrant acknowledgment. The quasi-experimental one-group repeated-measures design limits generalizability and causal inference. The absence of a control group makes it challenging to establish direct causal relationships between the intervention and observed improvements. Future research employing randomized controlled designs with diverse samples and comparison conditions (e.g., traditional HFS, standard clinical education) would provide more robust evidence of HFSECT’s specific contributions to learning outcomes.

The reliance on self-report measures introduces potential response bias and social desirability effects. Because debriefing, the proposed mediators, and problem-solving were all measured at T2, temporal ordering cannot be established, and the indirect effects should not be interpreted as causal mechanisms. The mediation model also relied on subscales from the same self-report instrument, which may have increased the observed associations through common-method variance. Baseline SLEI scores were high, which may have limited the scope for improvement, particularly for collaboration. In addition, the a priori power analysis was based on the repeated-measures comparison rather than the mediation model, so the indirect effects should be interpreted cautiously. Future studies should incorporate objective performance assessments during simulation scenarios, such as standardized checklists, expert evaluator ratings, or clinical competency examinations, to complement and validate self-reported data. Additionally, this study assessed only immediate post-intervention effects within 24 h of completion. Long-term skill retention and transfer to clinical practice remain unknown. Future research should include follow-up assessments at three, six, and twelve months after the intervention to determine whether the observed improvements are sustained and transferred to clinical practice. Multi-site studies across diverse educational contexts would further enhance external validity and inform implementation strategies for varied institutional settings.

## Data Availability

The datasets generated during the current study are available from the corresponding author on reasonable request.
